# Caries Experience before and after COVID-19 Restrictions: An Observational Study

**DOI:** 10.3390/jcm13041164

**Published:** 2024-02-19

**Authors:** Eduardo Guerreiro, João Botelho, Vanessa Machado, Luís Proença, José João Mendes, Ana Cristina Manso

**Affiliations:** 1 Egas Moniz Center for Interdisciplinary Research (CiiEM), Egas Moniz School of Health & Science, Caparica, 2829-511 Almada, Portugal; 2 Biomedicine Doctoral Program, Instituto de Ciências Biomédicas Abel Salazar da Universidade do Porto, 4050-313 Porto, Portugal; jbotelho@egasmoniz.edu.pt (J.B.); vmachado@egasmoniz.edu.pt (V.M.); lproenca@egasmoniz.edu.pt (L.P.); jmendes@egasmoniz.edu.pt (J.J.M.); cmanso@egasmoniz.edu.pt (A.C.M.)

**Keywords:** dental caries, caries experience, COVID-19, prevalence, pandemic, epidemiology, DMFT index

## Abstract

**Background:** The declaration of COVID-19 as a pandemic by the World Health Organization in 2020 led to the suspension of several clinical practices globally, including dentistry. This study investigates the impact of these restrictions on dental caries experience. **Methods:** A retrospective cross-sectional study was conducted at Egas Moniz University’s dental hospital in the Lisbon Metropolitan Area from June 2019 to June 2021. The study involved 3380 participants who were divided into two cohorts: after and before COVID-19 restrictions. Data collection included a questionnaire, full-mouth clinical examinations, and radiographs (panoramic X-rays, bitewings). **Results:** Before the COVID-19 restrictions, the prevalence of dental caries was 91.8%, with an average DMFT (decayed, missing, and filled teeth) index of 12.13. Post-restrictions, the prevalence decreased to 84.5%, with a DMFT index of 10.99. There was an increase in missing teeth and a decrease in decayed and filled teeth. Additionally, the frequency of toothbrushing declined among participants. **Conclusions:** The COVID-19 pandemic restrictions have significantly impacted dental caries experiences and oral health, highlighting a decrease in dental caries, but also a concerning reduction in oral hygiene practices. These results emphasize the importance of customized dental healthcare during public health emergencies to reduce impacts and maintain oral health.

## 1. Introduction

Dental caries, a prevailing disease worldwide, continues to affect a staggering 2.3 billion individuals who have their permanent teeth [1]. This condition is distinguished by an imbalanced state of the oral biofilm, brought about by consumable carbohydrates [1,2,3]. Because of variations in pH levels, there could be alternating periods of demineralization and remineralization. Should demineralization prevail, the integrity of tooth structures will suffer irreversible harm. If not treated, this sore will advance to the boundary between the dentine and the pulp, causing pain and discomfort. The incidence of dental caries is inevitably connected to a diminished perception of one’s quality of life and also imposes a substantial economic burden [4,5,6]. If dental caries is not appropriately managed, individuals afflicted by this condition may encounter difficulties with eating, tooth loss, toothaches, and delayed language development in children, as well as absenteeism from school and work [7,8].

As dental caries does not progress without the presence of bacteria in dental plaques, one of the most effective ways to prevent dental decay and gum disease is to remove plaque daily through brushing, flossing, and rinsing. The growth of dental plaque leads to the formation of caries and gum diseases, which, in turn, causes inflammation of the soft and hard tissues. This further worsens the loss of alveolar bone and ultimately leads to premature tooth loss [9].

In 2020, the World Health Organization (WHO) declared the COVID-19 outbreak a pandemic [1]. Uncertainty about its spread led health authorities worldwide to suspend certain clinical practices, including dentistry [3,4]. This suspension, in response to the pandemic, necessitated a shift in dental care focus. During the pandemic period, many dental offices and care institutions, adapting to the need for social isolation and new treatment protocols, were compelled to limit their services to only urgent and emergency cases [10,11].

Owing to these circumstances, the clinical care of patients has undergone remarkable changes, with an unknown impact on oral hygiene and oral health complications [5,6]. In this context, the dental office becomes a crucial setting for reinforcing good oral hygiene practices. Proper brushing and flossing methods, often overlooked in daily routines, may be effectively taught during routine check-ups, helping to mitigate any negative impacts on oral health due to altered clinical care [9,12].

The manner in which individuals consume food has experienced alterations, which encompasses an increase in the consumption of sugary foods and a decrease in oral hygiene. These changes could potentially result in anticipated complications concerning oral health, such as an increase in dental caries [9,13]. Dental caries remains one of the most prevalent oral diseases worldwide and is a public health challenge in the 21st century [14,15]. Dental caries contributes to the reduction of quality of life due to significant pain and discomfort [16] and is a marker of social disadvantages and health inequalities [13,17]. Another aspect to consider is how forced lockdowns negatively affected health determinants. Such negative effects of social distancing and the decreased support that occurred during lockdowns have been demonstrated in students’ mental health [7], stress and poor health in pregnancy [8], weight control [9], and even engaging in positive physical activities that ultimately may trigger undesirable behaviors [10].

However, despite this background, the impact of the COVID-19 pandemic on dental caries prevalence has been very little explored. We retrospectively analyzed a sample of first-incoming patients at a reference Portuguese university dental hospital to understand the effect of the COVID-19 pandemic restrictions on dental caries experience and dental health across demographic sectors. The null hypothesis (H0) of the presented study is that restrictions during the COVID-19 pandemic do not cause the deterioration of oral health in the studied population.

## 2. Materials and Methods

This study was conducted following the guidelines for strengthening the reporting of observational studies in epidemiology (STROBE) [11], in accordance with the Declaration of Helsinki of 1975, as revised in 2013, and was approved by the Institutional Review Board (or Ethics Committee) of Egas Moniz (ID no. 898 on 24 September 2020). The first appointment included gathering written informed consent from each participant.

### 2.1. Study Setting and Participants

This study was a retrospective, cross-sectional secondary analysis focusing on new patients at the Egas Moniz Dental Clinic, Almada, Portugal. The sample was obtained based on a temporal interval from June 2019 to June 2021 and a detailed database of incoming patients was used for the original data collection.

New patients underwent an initial assessment, including a self-reported health questionnaire, comprehensive oral examination, and radiographic evaluation with panoramic radiographs and bitewings. The questionnaire gathered key demographic details such as age, sex, education, employment, medical history, medication, smoking habits, and dental hygiene. Each patient underwent a customized evaluation and treatment plan. The initial observations were conducted by dental students under the guidance of clinical assistants from the diagnostic department who possess over 10 years of experience. These experienced professionals confirmed diagnoses and ensured the accuracy of the oral health assessments, providing an added layer of expertise and reliability to the process.

The inclusion criteria were age (patients must be 18 years or older) and consent (patients must provide written informed consent to participate in the study). The exclusion criteria are edentulism (patients who are completely edentulous will be excluded), incomplete data (patients with incomplete medical or dental records will be excluded), and special needs (patients with special needs who receive care in a specialized department will be excluded, as their conditions require different treatment approaches that are not the focus of this study).

Data were divided into two groups: group 1 (BCR)—before COVID-19 restrictions (June 2019 to early March 2020) and group 2 (ACR)—after COVID-19 restrictions (May 2020 to June 2021).

### 2.2. Dependent Variables

Caries experience was measured through the decayed, missing, and filled teeth (DMFT) index, a widely recognized and extensively used metric in dental research. Upon the initial visit, dental students, under the supervision of experienced clinical assistants from the diagnostic department with over ten years of expertise, conducted a comprehensive oral examination. This examination included recording the number of decayed (untreated), missing (due to caries), and filled teeth, constituting the initial DMFT score for each patient.

### 2.3. Independent Variables

We gathered data on sociodemographic characteristics and conducted a self-completed questionnaire prior to any clinical assessment. This methodology aims to mitigate the potential response influence.

In terms of independent variables, our study incorporated elements that illustrated health determinants and socio-demographic characteristics: age, gender, educational attainment level, professional status, and body mass index (BMI). Gender was divided into two sections: male and female. Age data were continuously calculated in years before categorization into the following groups: 18–24; 25–44; 45–64; ≥65. The classification of education levels was aligned with the guidelines set by the International Standard Classification of Education (ISCED) in their updated 2011 edition. Participants’ occupational statuses were categorized into four distinct groups for analysis: ‘student’ for those engaged in studentship or academic pursuits, ‘employed’ for actively working individuals, ‘unemployed’ for those currently without a job, and ‘retired’ for pension collectors or individuals no longer in the workforce. We evaluated oral hygiene practices based on the frequency of tooth brushing (2–3 times daily, once per day, 2–6 times weekly, or never), selection of a manual versus electric toothbrush, and the utilization of dental floss. This methodology of classifying the independent variables is shared by other studies that evaluated the same variables [18,19,20].

### 2.4. Statistical Analysis

Data analysis was conducted using IBM SPSS Statistics version 28.0 (IBM Corp., Armonk, NY, USA). We employed both descriptive and inferential statistical methods. The normality of data distribution was assessed using the Kolmogorov–Smirnov test, while Levene’s test evaluated variance homogeneity. The Kruskal–Wallis test was used for analyzing non-normally distributed data. When dealing with normally distributed data, the Student’s *t*-test or Welch test was applied, depending on variance homogeneity. The Mann–Whitney U test was utilized for non-normally distributed datasets. Throughout the study, a *p*-value of less than 0.05 (*p* < 0.05) was maintained as the threshold for statistical significance.

## 3. Results

### Participants Inclusion and Characteristics

**Figure 1:**
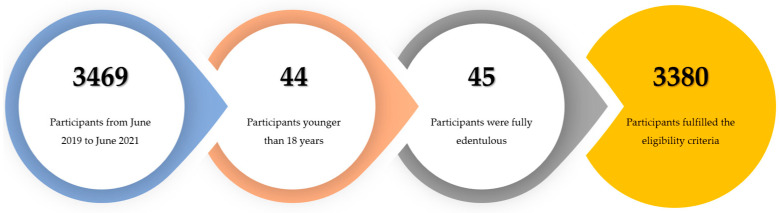
Participant inclusion flowchart.

Out of a total of 3469 incoming patients, 3380 (97.4%) fulfilled the eligibility criteria, while 89 participants were excluded from the study. Among the excluded individuals, 44 (1.3%) were younger than 18 years and 45 (1.3%) were edentulous (Figure 1).

Table 1 presents a comprehensive sociodemographic, health, and behavioral characterization of 3380 participants, divided into two periods: the before COVID-19 restrictions (BCR) group (2278 participants) and the after COVID-19 restrictions (ACR) group (1102 participants). The majority of the participants were female, constituting 59.4% overall, with a slight decrease from 60.1% in the BCR group to 56.8% in the ACR group. Overall, male participants accounted for 40.6%, with an increase from 39.9% in the BCR group to 43.2% in the ACR group. The average age of the participants was 43.21 years, with a minor decrease from the 43.36 years in the BCR group to 42.90 years in the ACR group. The age distribution was 21.3% in the 18–24 years category, 32.4% in the 25–44 years category, 32.2% in the 45–64 years category, and 14.2% in the ≥65 years category. Regarding education, 22.4% had no studies, 37.4% had completed elementary education, 26.9% had a middle-level education, and 3.3% had higher education, with a notable increase from 0.7% before the lockdown to 8.7% after the lockdown. Regarding occupation, 17.6% were students, 56.1% were employed, 10.4% were unemployed, and 15.9% were retired. In terms of body mass index (BMI), 3.6% fell below 18.5, 46.1% were within the 18.5–24.9 range, 34.1% in the 25.0–29.9 range, and 16.2% were 30.0 or above.

Table 2 provides detailed insights into oral healthcare practices and self-reported perceptions of oral health conditions. The use of manual toothbrushes was dominant at 88.6%, and remained constant across both periods. Electric toothbrush use was minor (10.9%), showing a slight increase from the BCR group to the ACR group.

There was a notable shift in toothbrushing frequency: 75.6% brushed 2–3 times daily, declining from 81.2% in the BCR group to 64.2% in the ACR group. Daily brushing was reported by 15.0%, while those brushing 2–6 times weekly increased significantly from 1.9% to 18.5% in the ACR group. A small fraction (2.1%) never brushed their teeth, increasing slightly from 1.5% in the BCR group.

Dental floss use declined from 38.0% in the BCR group to 26.3% in the ACR group, representing an overall decline of 34.2%. Participants’ perceptions of their teeth health were mostly stable across both periods, categorized as excellent (2.5%), very good (9.6%), good (43.3%), weak (29.3%), or very weak (15.2%). Similarly, gum health perceptions showed minor variations, with 4.0% rating it as excellent, 13.0% as very good, 52.2% as good, 24.2% as weak, and 6.5% as very weak. Gum bleeding was reported by 38.5% of participants, with a negligible difference between the two periods.

Table 3 illustrates a comparative review of indicators related to oral health in the BCR and ACR groups, focusing on decayed (DT), missing (MT), and filled teeth (FT). In addition, the overall decayed, missing, and filled teeth index measurements were also de-tailed. An interesting observation from the data is the reduction in the DMFT index across different demographics after the lockdown. Females exhibited a decline in their DMFT index from 12.38 to 11.01, while males observed a decrease from 11.74 to 10.96. This pattern may imply an enhancement in oral well-being or a change in dental care practices amid the period of lockdown. In terms of age, the youngest age group (18–24 years) encountered a slight elevation in their DMFT score from 8.25 to 9.76 in the ACR group, indicating potential negative effects of the lockdown on their oral health routines. Conversely, the DMFT scores in the other age groups decreased.

Educational background also plays a significant role. Individuals without formal education showed a noticeable reduction in their DMFT score from 14.34 to 11.48, while those with higher education showed a substantial decrease from 15.00 to 6.82. This finding highlights the influence of education on oral health maintenance. In terms of occupation, students and retired individuals experienced an increase in DMFT scores in the ACR group, whereas employed and unemployed groups saw a decrease.

Tooth brushing frequency correlated with DMFT scores, with those brushing 2–6 times weekly showing a significant decrease in their DMFT score in the ACR group. Furthermore, variations in dental caries experiences were observed across different BMI categories, suggesting a complex relationship between general health and oral hygiene.

**Figure 2:**
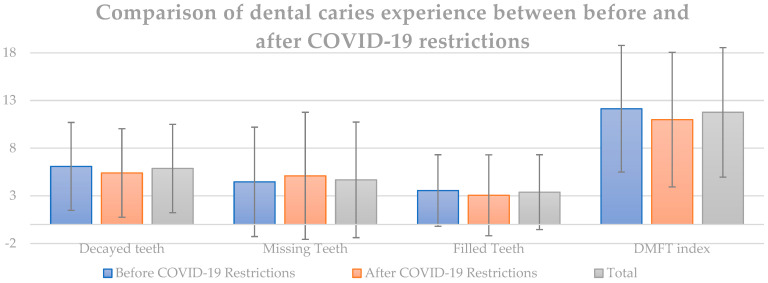
Comparison of dental caries experience between BCR group and ACR group.

As shown in Table 4 and Figure 2, there was a significant shift in the prevalence of dental caries following the COVID-19 restrictions among the 3380 participants. The percentage of individuals with dental caries (DCP) showed a notable decrease, moving from 91.8% in the BCR group to 84.5% in the ACR group. This decline signified a marked reduction in the proportion of samples affected by dental caries.

When examining the mean counts for various dental health indicators—decayed teeth, missing teeth, filled teeth, and the overall decayed, missing, and filled teeth index—several interesting trends emerged. The average count of decayed teeth decreased from 6.09 in the BCR group to 5.40 in the ACR group, indicating a reduction in the prevalence of this condition. However, the number of missing teeth increased slightly from 4.47 to 5.10. Additionally, there was a noticeable decrease in the mean count of filled teeth, from 3.56 in the BCR group to 3.06 in the ACR group. Correspondingly, the overall DMFT index, which combines these three measures, also showed a decrease, moving from 12.13 in the BCR group to 10.99 in the ACR group.

## 4. Discussion

This investigation retrospectively examined the incidence of dental caries in a Portuguese adult group, integrating both clinical evaluations and radiographic investigations during the phases before and after the COVID-19-induced restrictions. The objective of this study was to evaluate how Portugal’s oral health was impacted by this lockdown due to COVID-19, contrasting several oral wellness parameters between the two sets—those evaluated in the BCR group versus the ACR group—across varied demographic and behavioral factors. These specified parameters range from decayed teeth, missing teeth, filled teeth, and their amalgamated index, known as the decayed, missing, filled teeth index.

Notably, 89.4% of participants experienced some degree of exposure to caries. This high percentage of participants with at least one dental carie aligns with findings from previous studies [18,21].

Despite the high prevalence of dental caries in this population, the results indicated a decrease in the average number of dental caries and filled teeth in the ACR group, along with an increase in the average number of missing teeth. These results could be attributed to the reduced access to and utilization of dental-care services during the lockdown. During the pandemic, dental settings encountered significant restrictions, owing to their potential role in facilitating COVID-19 transmission, largely because of the nature of dental care procedures. Consequently, many countries have directed dental professionals to limit their services to urgent and emergency care, postponing elective treatments and implementing protocols to minimize transmission risks [22,23]. Routine dental checkups and treatments may have been deferred or canceled, leading to a decrease in the number of filled teeth. However, this could also account for the slight increase in missing teeth, as urgent dental issues might have necessitated extractions rather than restorative treatments [24,25,26]. Moreover, societal apprehension towards contracting COVID-19 has led to a significant decline in the need for dental services, even during emergencies [27,28,29]. The delay or evasion of regular diagnostic procedures and preventative measures is likely to contribute to the global increase in oral diseases [30]. This underlines the significance of sustaining self-care at home concerning oral hygiene when access to professional dentists is limited owing to such circumstances [31].

Another aspect potentially contributing to these results could be changes in oral hygiene practices during the lockdown. Altered daily routines may have affected oral care habits. It is plausible that people have more time to focus on oral health, potentially leading to improved hygiene practices. This improvement could contribute to the observed reduction in the decayed teeth count and overall DMFT index. While some studies indicated an increase in the frequency of home oral hygiene maintenance [31], others reported a decrease [32] or no significant change. Our results suggest an increase in the number of individuals who neglect regular tooth brushing and dental floss usage.

Additionally, other studies have noted an increase in dental caries following the lockdown. This increase could be associated with increased consumption of sugary and carbonated drinks during the pandemic, coupled with a decrease in the frequency and quality of oral hygiene [25,33].

Another possible reason for these findings could be related to the social distancing measures implemented during lockdown. Such measures might have reduced the transmission of bacteria, including cariogenic (cavity-causing) bacteria, thereby indirectly affecting the prevalence of dental caries [34].

Considering the demographic and behavioral factors, starting with sex, a reduction in the DMFT index was noticeable for both sexes following lockdown. Notably, there was an enhanced decline in tooth decay among women compared to men. However, it is important to note that previous research has identified sex-based differences in caries experiences [7]. Various factors, including societal norms, nutritional practices, dietary patterns, and hormonal variations, are known to contribute to sex-specific differences in the occurrence of carious lesions [35,36].

The data indicate a discernible escalation in DMFT index scores with age progression in both groups, underscoring a more pronounced occurrence of dental issues among older individuals. This trend is particularly evident with the highest DMFT scores observed in the BCR group for those aged 65 and older, registering at 15.39, and in the 45–64 age bracket in the ACR group, with scores of 11.30. This pattern aligns with the broader context of the pandemic, where preventive measures like social distancing and reduced face-to-face medical and dental consultations were essential to mitigate COVID-19 contagion risks, especially for the elderly [37,38]. Consequently, these restrictions likely exacerbated existing health issues [39]. Our findings corroborate this hypothesis, suggesting that the deficiencies in general and oral health among older individuals have intensified post-pandemic, reflecting the impact of limited healthcare access during this period.

The age group of 18–24 years showed a significant increase in DMFT scores during the lockdown, jumping from 8.25 to 9.76, indicating that this demographic was particularly affected. Consistent with existing research [18,35,40], our findings reaffirm that age is a key risk factor for dental health issues. Furthermore, this correlation might be influenced by various age-related factors such as dry mouth (xerostomia), the use of multiple medications (polypharmacy), functional and cognitive decline, and changes in the oral ecosystem over time [41,42].

All occupational groups showed a decrease in the DMFT index, with the most significant decrease observed among the students. These variations suggest that the impact of lockdown on dental health may differ according to lifestyle changes associated with occupation. It is well established in the literature that occupational environments significantly influence oral health [43,44,45].

Regarding the influence of education, people with higher educational levels (higher education) showed a significant decrease in the DMFT index in the ACR group, especially in the filled teeth category. Individuals with higher educational achievements often exhibit superior oral health. Research indicates that those with higher education generally possess a greater awareness of oral health and adhere more effectively to oral hygiene routines. Conversely, those with a less educational background are more inclined to neglect regular dental visits and care and frequently resort to emergency and treatment services rather than routine dental check-ups. Consequently, a positive correlation exists between higher educational levels and improved oral health outcomes [46,47,48].

In examining the impact of lockdown on dental health in relation to tooth brushing frequency, our results revealed changes. There was a 17% decrease in the number of individuals who brushed their teeth 1–3 times daily from before the COVID-19 restrictions to after the COVID-19 restrictions. Simultaneously, there was a 16.6% increase in the frequency of individuals brushing their teeth 1–6 times weekly over the same period. These results are consistent with the conclusion of a study aimed at evaluating the impact on oral health-related behaviors and practices of Portuguese and Spanish children during the COVID-19 lockdown [33].

Our research also identified a significant correlation between elevated body mass index and increased DMFT index scores, suggesting a potential interplay between dietary habits, overall health, and oral hygiene. We found that a higher BMI, a metric indicating body fat based on height and weight, was closely linked to the prevalence of dental caries. This trend is particularly visible among individuals classified as overweight or obese, who generally show higher levels of tooth decay, a finding that aligns with other studies [49,50,51]. The increased rate of tooth decay in these groups may be related to the consumption of diets high in sugary drinks and food [52]. Although our study points to a correlation between higher BMI and dental cavities, the complex relationship between obesity and oral health, including tooth decay, warrants further investigation. Notably, some studies have reported a contradictory association between lower instances of dental cavities and obesity [53,54], adding to the complexity of these interrelationships. This ambiguity is further supported by previous research conducted in Portugal that observed similar trends [18].

Individuals’ perceptions of their teeth health seem to correlate with their DMFT scores. Those with a perception of ‘weak’ or ‘very weak’ tooth health had higher DMFT scores, which increased in the ACR group. This may be related to the limited access to dental care [55].

The null hypothesis (H0) of the presented study is that restrictions during the COVID-19 pandemic do not cause the deterioration of oral health in the studied population. However, our findings compel us to refute the null hypothesis and embrace the alternative hypothesis, as there was a deterioration of oral health among the subjects being studied.

### Strenghts and Limitations

When assessing our research, it is important to consider both its merits and limitations. One significant limitation is the observational design of the study, which hinders our ability to determine causal relationships. However, it is particularly notable that this study involved a large number of participants. Furthermore, the fact that data collecting was predominantly undertaken by students poses a limitation, although this was somewhat counterbalanced by having all diagnoses validated by credentialed teachers with more than 10 years of experience. In addition, changes in dental procedures owing to the COVID-19 pandemic’s disruption of dental clinic functions have added another layer of restriction to this study. A noteworthy limitation is the lack of control over potential influencing factors such as fluoride exposure levels, salivary flow rate, or socioeconomic status, especially considering many patients’ hesitation about revealing their economic conditions.

There are certain constraints associated with the decayed, missing, and filled teeth index that we utilized in our investigation [56]. This measure combines decayed, missing, or filled teeth, but fails to distinguish between different causes of tooth loss other than dental caries, nor does it address the demand for oral healthcare intervention. However, proportions such as those representing decayed teeth out of total DMF count (D/DMF) and filled teeth counted against all accounted within the DMF set (F/DMF) offer an indication into unresolved treatment concerns and access to oral health services, respectively. The merit of our study lies in its radiographic verification of dental caries, which supports the credibility of our conclusions drawn from the results obtained during this research. Despite these caveats inherent within the analysis framework adopted by us, adherence has been ensured through globally acknowledged guiding principles [57,58,59], thereby reinforcing its significance and reliability across an extensive scholarly domain.

## 5. Conclusions

Considering the results and the examined population, we reject the null hypothesis of the present study. Our study reveals that the COVID-19 pandemic restrictions have unequally affected dental health across various demographic sectors. Key factors such as age, education, and professional status emerged as significant determinants in this disparity. Additionally, there was a notable shift in oral hygiene practices, particularly a marked reduction in the frequency of toothbrushing. While the average instances of dental caries and filled teeth decreased, there was a concerning increase in the number of missing teeth. The rise in missing teeth, coupled with the decline in positive oral hygiene habits during the confinement period, points to a potential deterioration in oral health within the population studied. This research underscores the necessity for more nuanced and targeted approaches in understanding and mitigating the disparities in oral health outcomes, especially in the context of pandemic-induced lifestyle changes.

## Figures and Tables

**Table 1:** Sociodemographic, health, and behavior characterization of the participants (*n* = 3380).

Variable	Total (*n* = 3380)	BCR Group (*n* = 2278)	ACR Group (*n* = 1102)
**Sex, % (*n*)**			
Female	59.4 (2007)	60.1 (1369)	56.8 (476)
Male	40.6 (1373)	39.9 (909)	43.2 (362)
**Age, mean**	43.21	43.36	42.90
**Age interval (years), % (*n*)**			
18–24	21.3 (719)	21.2 (484)	21.3 (235)
25–44	32.4 (1094)	32.3 (736)	32.5 (358)
45–64	32.2 (1088)	31.9 (727)	32.8 (361)
≥65	14.2 (479)	14.5 (331)	13.4 (148)
**Education, % (*n*)**			
No studies	22.4 (756)	24.1 (550)	18.7 (206)
Elementary	37.4 (1265)	37.7 (859)	36.8 (406)
Middle	26.9 (1247)	37.4 (853)	35.8 (394)
Higher	3.3 (112)	0.7 (16)	8.7 (96)
**Occupation, % (*n*)**			
Student	17.6 (594)	18.0 (411)	16.6 (183)
Employed	56.1 (1897)	55.8 (1271)	56.8 (626)
Unemployed	10.4 (350)	9.5 (217)	12.1 (133)
Retired	15.9 (539)	16.6 (379)	14.5 (160)
**BMI (Kg/m^2^) % (*n*)**			
<18.5	3.6 (120)	3.8 (86)	3.1 (34)
18.5–24.9	46.1 (1558)	44.5 (1013)	49.5 (545)
25.0–29.9	34.1 (1153)	34.4 (784)	33.5 (369)
≥30.0	16.2 (549)	17.3 (35)	14.0 (154)

Abbreviations: BMI—body mass index; *n*—number of participants; BCR—before COVID-19 restrictions; ACR—after COVID-19 restrictions. T-Student hypothesis tests were performed between both genders (*p* < 0.001) and age groups (*p* = 0.276). Statistical significance level was set at *p* < 0.05.

**Table 2:** Oral health care and self-reported perception about oral health condition descriptive data (*n* = 3380).

Variable	Total (*n* = 3380)	BCR Group (*n* = 2278)	ACR Group (*n* = 1102)
**Type of toothbrush, % (*n*)**			
Manual	88.6 (2993)	88.5 (2017)	88.6 (976)
Electric	10.9 (368)	10.8 (246)	11.1 (122)
**Toothbrush frequency % (*n*)**			
2–3 times/daily	75.6 (2555)	81.2 (1847)	64.2 (708)
1 time/daily	15.0 (507)	15.5 (352)	14.1 (155)
2–6 times/weekly	7.3 (248)	1.9 (44)	18.5 (204)
Never	2.1 (70)	1.5 (35)	3.2 (35)
**Dental Floss usage, % (*n*)**			
No	65.8 (2225)	62.0 (1413)	73.7 (812)
Yes	34.2 (1155)	38.0 (865)	26.3 (290)
**Teeth Heath Perception, % (*n*)**			
Excellent	2.5 (84)	2.5 (58)	2.4 (26)
Very good	9.6 (326)	9.4 (215)	10.1 (111)
Good	43.3 (1465)	43.7 (995)	42.6 (470)
Weak	29.3 (990)	29.4 (670)	29.0 (320)
Very Weak	15.2 (515)	14.9 (340)	15.9 (175)
**Gums Health perception, % (*n*)**			
Excellent	4.0 (136)	4.3 (97)	3.5 (39)
Very good	13.0 (440)	12.6 (287)	13.9 (153)
Good	52.2 (1766)	52.4 (1193)	52.0 (573)
Weak	24.2 (819)	24.1 (549)	24.5 (270)
Very Weak	6.5 (219)	6.7 (152)	6.1 (67)
**Gum bleeding. % (*n*)**			
No	61.5 (2078)	61.3 (1396)	61.9 (682)
Yes	38.5 (1302)	38.7 (882)	38.1 (420)
**Last dental visit, % (*n*)**			
<1 year	50.7 (1714)	51.5 (1174)	49.0 (540)
1–2 years	15.4 (522)	15.0 (342)	16.3 (180)
3–4 years	15.5 (524)	15.2 (347)	16.1 (177)
≥5 years	17.1 (578)	16.8 (383)	17.7 (195)
Never	1.2 (42)	1.4 (32)	0.9 (10)

Abbreviations: BCR, before COVID-19 restrictions; ACR, after COVID-19 restrictions.

**Table 3:** Comparison of dental caries experience between BCR and ACR groups, considering various variables (*n* = 3380).

Variable	BCR Group (*n* = 2278)				ACR Group (*n* = 1102)			
	DT	MT	FT	DMFT	DT	MT	FT	DMFT
**Sex**								
Female	5.88 (4.46) ^a^	4.62 (5.98) ^a^	3.87 (3.94) ^a^	12.38 (6.72) ^a^	5.39 (4.65) ^a^	5.39 (7.08) ^a^	3.27 (4.40) ^a^	11.01 (7.10) ^a^
Male	6.40 (4.8) ^b^	4.25 (5.34) ^a^	3.09 (3.39) ^b^	11.74 (6.50) ^b^	5.43 (4.63) ^a^	4.71 (6.03) ^a^	2.77 (4.00) ^a^	10.96 (6.98) ^a^
**Age interval** (**years**)								
18–24	4.91 (4.63) ^a^	0.32 (0.96) ^a^	2.10 (2.63) ^a^	8.25 (6.02) ^a^	4.50 (4.52) ^a^	3.08 (5.00) ^a^	1.39 (2.15) ^a^	9.76 (7.14) ^a^
25–44	6.77 (5.06) ^b^	2.21 (3.03) ^b^	3.83 (3.78) ^b^	11.24 (6.03) ^b^	5.83 (5.02) ^b^	3.26 (4.56) ^a^	2.99 (3.66) ^b^	11.20 (7.13) ^b^
45–64	6.30 (4.19) ^c^	6.73 (5.63) ^c^	4.56 (4.22) ^c^	14.13 (6.12) ^c^	5.75 (4.52) ^b^	6.48 (6.91) ^b^	4.08 (4.98) ^c^	11.45 (6.80) ^b^
≥65	5.82 (4.06) ^d^	10.63 (7.01) ^d^	2.89 (3.14) ^d^	15.39 (6.65) ^d^	4.97 (3.88) ^ab^	9.41 (9.32) ^b^	3.42 (5.21) ^b^	11.30 (7.15) ^b^
**Education**								
No studies	7.16 (5.07) ^a^	8.62 (7.29) ^a^	2.40 (2.98) ^a^	14.34 (6.96) ^a^	6.44 (4.78) ^a^	8.48 (8.48) ^a^	2.28 (3.38) ^a^	11.48 (6.61) ^a^
Elementary	6.22 (4.54) ^b^	3.72 (4.68) ^b^	3.49 (3.64) ^b^	11.49 (6.28) ^b^	5.44 (4.60) ^b^	4.29 (5.74) ^b^	3.67 (4.77) ^b^	11.59 (6.98) ^a^
Middle	5.22 (4.18) ^c^	2.43 (3.77) ^c^	4.40 (4.08) ^c^	11.29 (6.45) ^b^	5.09 (4.53) ^b^	4.59 (5.96) ^b^	3.29 (4.21) ^b^	11.14 (7.22) ^a^
Higher	8.56 (4.91) ^ab^	11.13 (6.32) ^a^	1.94 (3.24) ^a^	15.00 (6.61) ^c^	4.31 (4.60) ^b^	3.36 (6.30) ^c^	1.21 (2.63) ^c^	6.82 (6.17) ^b^
**Occupation**								
Student	4.27 (4.27) ^a^	0.41 (1.10) ^a^	2.35 (2.90) ^a^	8.28 (6.24) ^a^	4.33 (4.61) ^a^	3.83 (5.60) ^a^	1.38 (2.06) ^a^	9.55 (7.16) ^a^
Employed	6.44 (4.53) ^b^	3.87 (4.61) ^b^	4.22 (4.01) ^b^	12.16 (6.19) ^b^	5.80 (4.75) ^b^	4.48 (5.98) ^ab^	3.36 (4.32) ^b^	11.43 (7.01) ^b^
Unemployed	7.90 (5.21) ^c^	6.02 (6.98) ^c^	3.10 (3.82) ^ac^	13.84 (6.23) ^c^	5.45 (4.74) ^b^	5.28 (6.65) ^b^	3.33 (4.10) ^b^	10.82 (6.94) ^ab^
Retired	5.84 (4.23) ^d^	10.02 (6.79) ^d^	2.88 (3.09) ^c^	15.21 (6.70) ^d^	5.04 (3.88) ^b^	8.84 (8.75) ^c^	3.58 (5.33) ^b^	11.04 (7.01) ^b^
**Toothbrush frequency**								
2–3 times/daily	6.09 (4.63) ^a^	4.29 (5.65) ^a^	3.53 (3.76) ^a^	11.98 (6.64) ^a^	5.87 (4.52) ^a^	5.70 (6.79) ^a^	3.34 (3.88) ^a^	12.19 (6.74) ^a^
1 time/daily	6.16 (4.56) ^a^	5.07 (5.95) ^b^	3.56 (3.55) ^a^	12.63 (6.62) ^a^	5.72 (5.02) ^a^	5.49 (6.93) ^a^	3.34 (4.85) ^a^	10.84 (7.06) ^b^
2–6 times/weekly	5.18 (4.39) ^a^	6.07 (7.11) ^ab^	4.05 (4.20) ^a^	13.16 (6.06) ^a^	3.62 (4.21) ^b^	3.29 (6.01) ^b^	1.72 (4.04) ^b^	7.14 (6.68) ^c^
Never	6.40 (4.47) ^a^	6.03 (5.44) ^b^	4.49 (4.44) ^a^	13.31 (6.88) ^a^	4.97 (5.27) ^ab^	1.80 (2.86) ^b^	3.97 (7.27) ^a^	9.89 (7.12) ^ab^
**BMI** (**Kg/m^2^**)								
<18.5	5.56 (4.71) ^ac^	2.64 (497) ^a^	2.57 (2.29) ^a^	10.72 (6.91) ^a^	3.35 (4.13) ^a^	4.26 (6.11) ^a^	1.38 (1.46) ^a^	9.21 (6.91) ^a^
18.5–24.9	5.63 (4.76) ^a^	3.38 (5.43) ^a^	3.60 (3.81) ^a^	11.23 (6.62) ^a^	5.13 (4.59) ^b^	4.27 (6.11) ^a^	2.78 (3.94) ^a^	10.42 (7.08) ^a^
25.0–29.9	6.41 (4.60) ^bc^	5.25 (5.72) ^b^	3.71 (3.91) ^a^	12.76 (6.51) ^b^	6.09 (4.76) ^b^	5.57 (6.67) ^ab^	3.34 (4.44) ^ab^	11.85 (7.05) ^b^
≥30.0	6.72 (4.85) ^b^	6.12 (6.00) ^c^	3.35 (3.69) ^a^	13.47 (6.51) ^b^	5.18 (4.40) ^b^	7.11 (8.03) ^b^	3.75 (4.99) ^a^	11.34 (6.78) ^ab^
**Teeth Heath Perception**								
Excellent	4.60 (4.13) ^a^	2.03 (4.38) ^a^	2.02 (2.59) ^a^	8.76 (6.82) ^a^	3.54 (3.58) ^a^	4.69 (7.86) ^a^	3.23 (4.18) ^a^	10.31 (5.87) ^a^
Very good	6.06 (5.08) ^b^	2.06 (3.69) ^a^	2.63 (3.34) ^a^	10.07 (6.29) ^a^	4.83 (5.08) ^a^	4.18 (5.89) ^a^	1.88 (2.99) ^a^	9.95 (6.71) ^a^
Good	5.87 (4.63) ^b^	3.79 (5.50) ^b^	3.47 (3.69) ^b^	11.55 (6.74) ^b^	5.20 (4.61) ^a^	3.82 (5.44) ^ab^	2.94 (3.79) ^ab^	10.94 (7.14) ^a^
Weak	6.27 (4.40) ^b^	5.56 (5.97) ^c^	3.91 (3.83) ^c^	13.20 (6.30) ^c^	5.99 (4.50) ^b^	6.40 (7.56) ^ac^	3.31 (4.36) ^ab^	11.45 (7.09) ^a^
Very Weak	6.64 (4.67) ^c^	6.26 (6.20) ^c^	3.97 (3.99) ^c^	13.56 (6.46) ^c^	5.52 (4.70) ^b^	6.82 (7.36) ^ac^	3.67 (5.55) ^ab^	11.03 (7.05) ^a^

Data are presented as mean (standard deviation). Different letters indicate statistically different mean values (Kruskal-Wallis test, *p* < 0.05). Abbreviations: BMI, body mass index; DT, decayed teeth; MT, missing teeth; FT, filled teeth; DMFT, decayed, missing, and filled teeth index; *n* = number of participants; BCR, before COVID-19 restrictions; ACR, after COVID-19 restrictions. The statistical significance level was set at *p* < 0.05.

**Table 4:** Comparison of dental caries experience between BCR and ACR groups (*n* = 3380).

Variable	DCP (%; *n*)	DT	MT	FT	DMFT
**BCR group**	91.8 (2092) ^a^	6.09 (4.61) ^a^	4.47 (5.73) ^a^	3.56 (3.75) ^a^	12.13 (6.64) ^a^
**ACR group**	84.5 (931) ^b^	5.40 (4.64) ^b^	5.10 (6.66) ^a^	3.06 (4.24) ^b^	10.99 (7.05) ^b^
**Total**	89.4 (3023)	5.87 (4.63)	4.68 (6.06)	3.39 (3.92)	11.76 (6.79)

Data are presented as mean (standard deviation). Different letters indicate statistically different mean values among the variables (Mann–Whitney test, *p* < 0.05). Abbreviations: DCP, dental caries presence; DT, decayed teeth; MT, missing teeth; FT, filled teeth; DMFT, decayed, missing, and filled teeth index; *n* = number of participants; BCR, before COVID-19 restrictions; ACR, after COVID-19 restrictions. The statistical significance level was set at *p* < 0.05.

## Data Availability

The entirety of the data compiled and examined throughout this study can be found within this document. Any additional queries should be directed towards the author in charge of correspondence.
